# Shaping Up the Tumor Microenvironment: Extracellular Vesicles as Important Intermediaries in Building a Tumor-Supportive Cellular Network

**DOI:** 10.3390/cancers15020501

**Published:** 2023-01-13

**Authors:** Amod Sharma, Ajay Pratap Singh, Seema Singh

**Affiliations:** 1 Mitchell Cancer Institute, University of South Alabama, Mobile, AL 36604, USA; 2 Department of Pathology, University of South Alabama, Mobile, AL 36617, USA; 3 Department of Biochemistry and Molecular Biology, College of Medicine, University of South Alabama, Mobile, AL 36688, USA

A tumor is not just comprised of cancer cells but also a heterogeneous group of infiltrating and resident host cells, as well as their secreted factors that form the extracellular matrix [1,2,3]. It is extremely important for the cancer cells to build a supportive environment around them that sustains their growth and protects them from internal immune attack and external challenges, including radiation, immune-, and chemotherapy. In addition, this supportive tumor microenvironment (TME) also provides the passageways for the tumor cells to flee when they encounter tough situations and find newer homing sites (metastasis) [3,4]. The formation of supportive TME requires effective communication of cancer cells with the host cells to allow their recruitment, conditioning, and expansion. Cancer cells use various modes of intercellular communications, including direct cell-to-cell contact through membrane-bound ligand–receptor interactions, gap junctions, and tunneling nanotubes. In addition, they also release soluble factors, such as hormones and cytokines, which allow near- and far-distance communication via paracrine or endocrine signaling [5].

In recent decades, a novel mode of intercellular communication via extracellular vesicles (EVs) has gained significant attention, especially in disease conditions [6]. EVs are membranous structures of varying sizes shed by nearly all cell types [7,8,9]. In general, smaller size EVs (30–100 nm diameter) are of endosomal origin and referred to as ‘exosomes’, while medium-sized EVs (100 to 1000 nm diameter) are pinched off directly from the plasma membrane and are known as ‘microvesicles’. Large EVs (1000 to 5000 nm diameter) originate from membrane-blebbing during the apoptotic cell death and are thus referred to as ‘apoptotic bodies’. Significant overlap; however, has been reported in size distribution and marker profiles among different EV subtypes, especially under disease conditions [10]. EVs carry bioactive cargo such as proteins, mRNA, miRNA, and DNA from the donor cells, and their uptake by the recipient cells alters their functions and behavioral phenotypes. The shedding of EVs and their composition vary between cell types and are greatly affected by environmental exposure [10,11].

It is now well-established that EVs influence a wide range of processes that support tumor development. A recent review by Mittal et al. published in *Cancers* beautifully captures the role of EVs in immune regulation [12]. The immune system is our major defense against infections and injuries. However, cancer cells not only dodge this strong defense system but also hijack and use it for their benefit [13,14]. Virchow postulated that cancer develops as the product of unresolved inflammation [15]. In fact, chronic inflammation is a key hallmark of cancer and plays an important role in cancer development. The question, however, is how do cancer cells trick this important defense mechanism? Years of research have provided several clues, and the field continues to evolve. Cancer cells have been demonstrated to suppress the antitumor immune response by impairing the antigen presentation and inhibiting the natural killer cells, cytotoxic T cells and phagocytic cells [16,17]. It is also shown that cancer cells alter the differentiation of immune cells from immunoprotective to immunosuppressive states, such as Treg cells, M2 macrophages, and myeloid-derived suppresser cells (MDSCs) through the release of anti-inflammatory cytokines [18,19].

After recognizing that EVs contain bioactive material and can alter the phenotype of the recipient cells, researchers began to investigate their roles in immune responses as well. Mittal et al. have reviewed several studies describing how the EVs released from tumor cells transmit signals to various immune cell types to affect their proliferation, survival, and metabolism [12]. They describe both the immune-suppressive and immune-stimulatory functions of tumor-derived EVs. In one study, prostate tumor cell-derived EVs are shown to downregulate NKG2D expression on NK cells and CD8^+^ T cells, which helps in immune evasion [20]. Tumor-derived EVs expressing Fas ligand can also mediate the apoptosis of CD8^+^ T cells [21]. Similarly, EVs can also carry PD-L1 on their surface and cause immune suppression [22]. In other reports, tumor-derived EVs are shown to alter immune function by affecting gene expression or by delivering regulatory microRNAs to T cells [23]. Interestingly, several studies also demonstrate that tumor-derived EVs can also stimulate immune response; however, underlying molecular mechanisms for such contrasting responses are unclear. It appears that other factors within the TME, tumor-specific alterations, and multi-directional crosstalk among tumor and various stromal cells affect the final outcome [24,25,26].

In addition to acting on the immune cells, EVs shed from the tumor cells can also affect the functions of other stromal cells and promote the stress-coping behavior of tumor cells. In a recent study, we have shown that EVs from chemotherapy-challenged pancreatic cancer cells promote chemoresistance by promoting ROS detoxification and the miR-155-mediated suppression of drug-metabolizing enzymes [27]. Hypoxia, which has been shown to promote tumor aggressiveness and therapy resistance, also alters the release and composition of EVs from the tumor cells. The EVs derived from hypoxic cells not only promote the hypoxia adaptiveness of the uninitiated cancer cells but also confer chemoresistance [10,28,29]. The role of tumor-derived EVs in pre-metastatic niche formation and organotropism has also been established [30,31]. Similarly, EVs derived from gastric cancer cells were shown to promote peritoneal metastasis by disrupting the mesothelial barrier and causing peritoneal fibrosis [32].

Multiple immune cell types have also been shown to shed EVs and utilize them to communicate with the tumor and other neighboring cells in the TME. EVs from B cells carry surface receptors, MHC proteins, co-stimulatory molecules, and antigens that can activate antigen-specific immune responses [33]. Similarly, EVs derived from dendritic cells can activate antigen-specific T cell-mediated toxicity [34]. T cells also shed EVs that carry bioactive cargo and exert response to the donor cells. A recent study showed that EVs derived from engineered CAR-T cells could induce potent anti-tumor responses with minimal toxicity [35]. Activated NK cells also produce EVs expressing cytotoxic proteins that can cause caspase-mediated tumor cell apoptosis, suggesting their potential utility as an immunotherapeutic treatment [36,37].

In addition to their functional impact, Mittal et al. also discuss the translational potential of EVs in biomarker development and their utility as efficient drug delivery carriers [12]. A significant aspect of EVs as tools for biomarker development is that they can potentially be traced back to the donor cells, which increases their specificity. Furthermore, unlike circulating tumor DNA/RNA, EVs are highly stable in biological fluids and keep the encapsulated biomarker material protected from lysis. In addition, EVs carry multiple tumor-specific molecules that can help monitor the disease state since the composition of EVs varies depending on their environmental exposures [38,39,40,41]. In a recent study, we detected high-frequency mitochondrial DNA mutations in EVs isolated from the serum of pancreatic cancer patients. These EVs were highly enriched for mitochondrial DNA and a mitochondrial membrane lipid, suggesting their mitochondrial origin [42]. Being a natural carrier of bioactive molecules, EVs can also be exploited as an efficient drug delivery system [43,44]. EVs from different cell types can vary in drug-loading capacity and antitumor efficacy [7]. Further, efforts have also been made to increase the immunostimulatory response of EVs [45,46,47]. In some studies, the inhibition of tumor exosome biogenesis has also been investigated as a potential anti-cancer strategy [48,49].

In summary, EVs have significant potential to revolutionize cancer care. They not only play multifaceted roles in tumor pathobiology but are also a rich resource for biomarker development. However, we need more refined EV isolation strategies to improve their yield and purity since most clinical samples are not available in large volumes. In addition, characterizing cell-specific markers carried by EVs is also an important task while simultaneously developing clinically feasible techniques for their detection. We have started to gain insights into the pathways that regulate EV biogenesis, shedding, and loading of the material within them. Such information could help us develop strategies to target EVs for preventive and therapeutic interventions. The utility of EVs as drug carriers is being investigated at a rapid pace by improving drug-loading strategies as well as engineering the EVs to improve targeted delivery and uptake, and to impart new bioactive properties for therapeutic enhancement. Once thought of as trash bags carrying waste cellular material, EVs have come a long way. We hope that this decade will see EV-based approaches in clinics to manage cancer more effectively, thus improving the life expectancy of patients diagnosed with lethal malignancies.
